# The Neurotypical Assumption in Consumer Research: Rethinking Consumer Heterogeneity Through Neurocognitive Diversity

**DOI:** 10.3390/bs16101725

**Published:** 2026-09-23

**Authors:** Polona Dakič, Armand Faganel

**Affiliations:** Faculty of Management, University of Primorska, 6000 Koper, Slovenia

**Keywords:** consumer behavior, consumer theory, consumer heterogeneity, neurocognitive diversity, cognitive neuroscience, theory development

## Abstract

Understanding why individuals perceive, interpret, and respond differently to the same environmental stimuli represents one of the central questions in the behavioral sciences. Across the psychology, cognitive science, neuroscience, and behavioral decision research, substantial effort has been devoted to explaining the mechanisms underlying human attention, perception, learning, judgment, and decision making. Although these disciplines increasingly acknowledge considerable individual variability in cognitive functioning, theoretical models have often emphasized generalizable cognitive mechanisms, raising questions about the conditions under which behavioral theories generalize across populations. This article introduces the neurotypical assumption as a heuristic concept for identifying an under-specified scope condition in consumer research: the extent to which established theoretical mechanisms and empirical regularities generalize across neurocognitive variation. Rather than presuming that consumer theories are inherently neurotypical, the concept directs attention to whether neurocognitive characteristics constitute theoretically meaningful sources of heterogeneity that may qualify particular consumer-theory mechanisms and predictions under particular conditions. Building on insights from consumer research, cognitive neuroscience, developmental psychology, and neurodiversity scholarship, we develop a conceptual framework that examines how neurocognitive variation may interact with marketplace environments and potentially qualify processes of attention, information processing, learning, judgment and decision making, and marketplace interaction. The framework therefore positions neurocognitive variation as a potential source of mechanism-relevant consumer heterogeneity whose theoretical significance requires empirical examination. This article makes three contributions. First, it introduces the neurotypical assumption as a heuristic for identifying potentially under-specified neurocognitive scope conditions in consumer theory. Second, it integrates interdisciplinary evidence to examine whether documented neurocognitive variation provides a theoretically plausible basis for specifying additional sources of consumer heterogeneity. Third, it develops an integrative framework that positions neurocognitive variation within person–environment interactions and identifies testable research directions for examining when and how neurocognitive characteristics may qualify established consumer-theory mechanisms and predictions.

## 1. Introduction

Consumer research provides an informative context in which these broader questions become particularly visible. Because marketplace decisions involve attention, perception, information processing, learning, and judgment, consumer behavior has long served as an applied domain for studying human cognition. Existing theories have generated sophisticated explanations for differences in consumer behavior by emphasizing motivation, preferences, knowledge, goals, emotions, identities, and contextual influences. However, the neurocognitive scope of these explanations has generally remained under-specified, leaving open the question of whether the cognitive mechanisms supporting them operate comparably across neurocognitive variation.

Understanding consumer heterogeneity has long been a central objective of marketing and consumer research because differences among consumers provide the foundation for market segmentation, targeting, positioning, product development, customer experience management, and personalization strategies. Consequently, explaining why consumers respond differently to similar marketplace stimuli is not only a theoretical challenge but also essential for developing more accurate consumer theories and more effective marketing strategies. Although existing consumer theories have generated substantial insights into these differences, they have primarily focused on variation in psychological, social, and contextual characteristics rather than potential variation in the neurocognitive processes through which marketplace information is processed.

More broadly, behavioral science has increasingly questioned the extent to which findings derived from relatively homogeneous populations can be generalized across diverse human populations (Henrich et al., 2010). Similar concerns have emerged across cognitive science and psychology, where growing attention has been devoted to identifying the boundary conditions under which established theories remain applicable. These developments provide an important foundation for examining under-specified scope conditions within consumer theory.

Despite their conceptual diversity, however, these theories have primarily conceptualized consumer heterogeneity through variation in psychological, social, and contextual factors. The extent to which neurocognitive characteristics qualify the mechanisms through which consumers attend to, interpret, learn from, and respond to marketplace information has been less explicitly specified. Consequently, an important open question is whether variation in neurocognitive processes contributes to consumer behavior beyond, or in interaction with, established psychological, social, and contextual sources of heterogeneity. This historical emphasis on generalizable cognitive mechanisms reflected the dominant assumptions of cognitive science during the formative decades of consumer research. Consequently, consumer heterogeneity was primarily conceptualized as variation in psychological and contextual characteristics rather than variation in underlying neurocognitive processes. Contemporary behavioral science increasingly emphasizes that cognition emerges from interactions among neurobiological, psychological, social, and environmental processes rather than from isolated and universally uniform mechanisms (Cacioppo & Decety, 2011).

Accumulating evidence demonstrates meaningful individual variation in executive functioning and cognitive flexibility (Diamond, 2013; Friedman & Miyake, 2017), internal predictive models (Wang et al., 2024), and the functional organization of large-scale brain networks (Dworetsky et al., 2024). These findings document meaningful individual variation in cognitive functioning and organization, although they do not by themselves establish fundamentally different cognitive architectures or demonstrate corresponding differences in consumer behavior.

These scientific developments raise an important theoretical question for consumer research. If consumers differ not only in their motivations, preferences, and experiences, but also in the organization of the cognitive mechanisms through which marketplace information is processed, to what extent do existing theories adequately explain the full range of consumer behavior? More specifically, to what extent do established consumer-theory mechanisms and predictions generalize across neurocognitive variation, and under what conditions might neurocognitive characteristics meaningfully qualify them?

As a conceptual article, the present study does not aim to provide a systematic or exhaustive review of the literatures it integrates. Instead, the literature was selected and synthesized to support theory development across three complementary purposes: to identify influential consumer-research traditions in which cognitive mechanisms are central to theoretical explanation; to establish evidence of systematic neurocognitive variation in processes relevant to these theories; and to examine whether such variation provides a theoretically plausible basis for specifying additional scope conditions of established consumer-theory predictions. The analysis therefore integrates foundational and representative work from consumer research with relevant evidence from cognitive neuroscience, developmental psychology, and neurodiversity scholarship. Throughout the article, evidence documenting neurocognitive variation is distinguished from consumer-specific evidence and from theoretical predictions that require future empirical testing.

To address this question, this article introduces the neurotypical assumption as a heuristic concept for examining the under-specified neurocognitive scope of consumer theory. We define the neurotypical assumption not as an empirically established premise that consumer theories explicitly rely on neurotypical cognition, but as a heuristic for identifying cases in which the extent to which theoretical mechanisms and empirical regularities generalize across neurocognitive variation remains unspecified. Historically, influential consumer theories have sought to identify mechanisms capable of explaining behavior across consumers and contexts, while neurocognitive characteristics have rarely been specified as potential conditions affecting the operation or generalizability of these mechanisms. From this perspective, the neurotypical assumption does not denote an empirically established property of consumer theory, but provides a conceptual lens for examining whether neurocognitive variation constitutes a theoretically meaningful source of heterogeneity that may qualify established consumer-theory predictions. The term “neurotypical assumption” is therefore used as a heuristic label for this theoretical under-specification, rather than to suggest that consumer theories explicitly or implicitly assume a neurotypical cognitive architecture. The term identifies a question about theoretical scope—not a claim about the assumptions on which existing consumer theories were originally constructed.

The issue is instead one of specified theoretical scope: when neurocognitive characteristics are neither measured nor incorporated into theoretical models, the extent to which established mechanisms and empirical effects generalize across neurocognitive variation remains an open empirical question. Neurocognitive diversity may therefore represent a previously underexamined source of heterogeneity that qualifies particular theoretical relationships under particular conditions, rather than a universal boundary condition applied uniformly to consumer theory as a whole.

Building on developments in consumer behavior, cognitive neuroscience, developmental psychology, and neurodiversity scholarship, this article develops a conceptual framework that brings neurocognitive variation more explicitly into theories of consumer heterogeneity. Rather than proposing a new general theory of consumer behavior, the framework examines whether and under what conditions neurocognitive characteristics may qualify established theoretical relationships. In doing so, it complements existing research on consumer individual differences, cognitive capacities, vulnerability, disability, and person–environment heterogeneity. Prior research on disabled consumers has shown that marketplace vulnerability and exclusion may emerge not simply from individual impairment, but from interactions between consumer characteristics and inaccessible market structures, information, and practices (Eskytė, 2019). The present framework builds on this person–environment perspective by focusing specifically on whether variation in the cognitive processes through which consumers engage with marketplace environments may qualify established consumer-theory mechanisms and predictions.

An emerging stream of consumer and marketing scholarship has begun to address neurodiversity more directly. Go Jefferies and Ahmed (2022), for example, examined neurodiversity within a marketing context and demonstrated heterogeneity in how neurodiversity is experienced and represented among consumers. More recently, Go Jefferies et al. (2025) adopted a neurodiversity perspective on marketplace inclusion, emphasizing mismatches between consumers’ characteristics and marketplace environments. Consumer-specific empirical studies have also begun to document neurodivergence-related variation in marketplace contexts, including associations between autism-spectrum characteristics and exploratory consumer behavior (Baas & van Raaij, 2010) and differences in responses to high-pressure advertising among autistic and non-autistic consumers (Verrette & Bentley, 2025). Together, these studies demonstrate growing recognition of neurodiversity within consumer scholarship. However, this literature remains emergent and has primarily focused on consumer experiences, inclusion, segmentation, or particular behavioral outcomes rather than systematically examining neurocognitive variation as a potential scope condition of established consumer-theory mechanisms. The present framework builds on this emerging literature by addressing this latter theoretical question. This article makes three contributions to consumer research. First, it introduces the neurotypical assumption as a heuristic concept for making an under-specified neurocognitive scope condition in consumer theory explicit. Second, it integrates insights from consumer research, cognitive neuroscience, developmental psychology, and neurodiversity scholarship to distinguish neurocognitive variation from established sources of consumer heterogeneity and to identify potential mechanisms through which such variation may qualify consumer-theory predictions. Third, it develops an integrative conceptual framework that positions neurocognitive variation within person–environment interactions and identifies conditions under which it may qualify established consumer-theory mechanisms and predictions.

The remainder of the article examines the historical and interdisciplinary foundations of neurocognitive under-specification, develops the proposed conceptual framework, and concludes by considering its theoretical, methodological, and research implications.

## 2. The Neurotypical Assumption as an Under-Specified Scope Condition in Consumer Theory

Consumer research has long sought to develop theoretical explanations that identify regularities in marketplace behavior while also accounting for meaningful sources of consumer heterogeneity. Influential traditions have progressively incorporated variation in motivation, knowledge, expertise, affect, identity, culture, and situational context to explain why consumers may respond differently to similar marketplace stimuli. This under-specification can be illustrated across several influential traditions in consumer research. For example, information-processing models explain consumer choice through mechanisms involving information acquisition, processing, memory, and choice (Bettman, 1979), while research on consumer expertise demonstrates how knowledge and experience shape cognitive effort, cognitive structure, analysis, elaboration, and memory (Alba & Hutchinson, 1987). Research on visual attention further demonstrates how characteristics of advertising stimuli influence attentional allocation (Pieters & Wedel, 2004). These traditions explicitly recognize important sources of consumer heterogeneity, while the cited theoretical and empirical formulations do not explicitly specify whether neurocognitive variation qualifies the proposed mechanisms or their behavioral predictions.

Our argument therefore concerns a more specific question: whether neurocognitive characteristics have been comparably articulated as potential scope conditions of consumer-theory predictions. General theories necessarily abstract from some forms of individual variation, and the absence of a variable from a theoretical model does not by itself constitute an assumption about that variable. We use the term neurotypical assumption more narrowly to identify a recurring under-specification: where theories propose cognitive mechanisms or empirical regularities without explicitly considering whether their operation varies with neurocognitive characteristics, their neurocognitive scope remains unspecified. The theoretical relevance of this omission must consequently be demonstrated rather than presumed, by identifying cases in which neurocognitive variation can plausibly alter the mechanisms or boundary conditions of established consumer effects.

Consumer research has extensively examined cognitive individual differences, including variation in knowledge, expertise, processing capacity, cognitive style, self-regulation, and responses to cognitive load. These research traditions demonstrate that differences in cognition are not absent from consumer theory. However, they typically examine particular abilities, traits, states, or task-related constraints rather than asking more broadly whether variation in the organization and operation of cognitive processes systematically qualifies established consumer-theory predictions. The distinction is therefore not between consumer literature that recognizes cognitive heterogeneity and one that does not. Rather, the present framework asks whether neurocognitive variation provides an additional level of explanation that can complement established individual-difference approaches by specifying when differences in underlying cognitive processes interact with marketplace environments to alter theoretically expected outcomes. Theoretical scope conditions often become more visible as advances in adjacent disciplines identify previously underexamined sources of variation. Rather than invalidating established theories, scientific progress frequently involves specifying additional conditions under which theoretical mechanisms and predictions may vary. From this perspective, recent developments in cognitive neuroscience provide an opportunity to examine whether neurocognitive variation represents one such under-specified source of heterogeneity in consumer theory.

The relevant question is therefore not whether established theories were developed for a demonstrably neurotypical population, but whether their proposed mechanisms and empirical regularities generalize across neurocognitive variation. Where neurocognitive characteristics systematically alter a focal mechanism or theoretical relationship, they may constitute a meaningful boundary condition; where they do not, existing theoretical predictions may generalize without such qualification. Neurocognitive variation may offer a further source of such qualification, but its incremental theoretical value depends on demonstrating that it explains variation not adequately captured by established constructs such as knowledge, expertise, cognitive capacity, psychological traits, or contextual constraints. The relevant theoretical question is therefore not simply whether consumers differ neurocognitively, but whether and when such variation changes the operation of mechanisms underlying established consumer effects.

Its theoretical usefulness depends on moving beyond the observation that neurocognitive variation exists and specifying where such variation is expected to alter established consumer mechanisms or predictions. This requires distinguishing evidence that documents neurocognitive variability from evidence that directly demonstrates variation in consumer phenomena, as well as from theoretical predictions that remain to be empirically tested. The following sections therefore first clarify the conceptual basis of neurocognitive variation and then examine how it may become theoretically consequential for specific consumer processes and marketplace contexts.

## 3. Historical Foundations of Neurocognitive Under-Specification in Consumer Theory

The under-specification of neurocognitive scope conditions can be understood partly in relation to the intellectual foundations of consumer research. As the field developed, it drew extensively on cognitive psychology and related behavioral sciences to explain processes such as attention, information processing, memory, judgment, and decision making (Neisser, 1967; Anderson, 1983). This intellectual tradition strongly influenced consumer research, particularly through information-processing approaches to consumer decision-making (Bettman, 1979; Bettman et al., 1998). Theoretical progress within these traditions depended on identifying general mechanisms that could organize and predict behavior across individuals and contexts. This emphasis on generalizable cognitive processes was theoretically productive and contributed substantially to the development of consumer theory. At the same time, questions concerning whether these mechanisms operate similarly across different neurocognitive characteristics were rarely central to the theoretical scope of these traditions. Methodological conventions further reinforced the emphasis on identifying generalizable consumer effects and on specifying the conditions under which experimental findings could be extended beyond particular samples and settings (Calder et al., 1981; Lynch, 1982). Experimental research typically relies on standardized tasks, controlled stimuli, and aggregate comparisons designed to isolate theoretically relevant relationships while minimizing extraneous variation. These practices have been essential to cumulative theory development, but they also mean that sources of heterogeneity not explicitly measured or manipulated may remain outside the specified scope of empirical models. Neurocognitive characteristics represent one such potentially relevant source of variation. Importantly, this does not establish that existing samples were neurotypical or that observed effects apply only to neurotypical consumers; rather, when neurocognitive characteristics are not assessed, the extent to which observed relationships generalize across such variation remains empirically undetermined.

Recent advances in cognitive neuroscience, developmental psychology, and neurodevelopmental science provide a basis for examining neurocognitive variation as a potential source of theoretically relevant heterogeneity. Contemporary evidence documents systematic individual variation in attentional regulation, executive functioning, cognitive flexibility, and the functional organization of large-scale brain networks (Diamond, 2013; Friedman & Miyake, 2017; Dworetsky et al., 2024). Predictive-processing accounts further characterize cognition as relying on generative models through which the brain anticipates and interprets incoming information (Friston, 2010; Clark, 2016), with recent evidence also pointing to meaningful individual differences in such internal models (Wang et al., 2024). This evidence documents the existence of neurocognitive variation, but it does not by itself demonstrate that such variation modifies consumer-theory mechanisms or marketplace outcomes. Rather, it provides the evidentiary basis for asking when neurocognitive characteristics interact with consumer tasks and marketplace environments in ways that qualify established theoretical predictions.

## 4. Conceptualizing Neurocognitive Diversity and Cognitive Architecture

Recent advances in cognitive neuroscience, developmental psychology, and neurodiversity scholarship have increasingly emphasized systematic variation in human cognition. In this article, we use neurocognitive diversity as an umbrella term for variation across individuals in the functioning and organization of cognitive processes, including attention, executive functioning, learning, reward processing, sensory processing, and related mechanisms. The concept is dimensional: it is not restricted to individuals with diagnosed neurodevelopmental conditions and does not imply that consumers can be divided into two cognitively homogeneous groups of “neurotypical” and “neurodivergent” individuals. Rather, neurocognitive characteristics may vary both within and across diagnostic categories and across the broader population. This dimensional perspective provides the basis for examining whether particular neurocognitive characteristics become theoretically consequential in particular consumer contexts. For consumer research, these developments have important theoretical implications. Traditional consumer theories have typically focused on generalizable cognitive mechanisms while leaving the neurocognitive scope of these mechanisms comparatively under-specified. Although established models recognize substantial variation in consumer knowledge, motivation, expertise, involvement, and other individual characteristics, they have less frequently examined whether the cognitive processes underlying consumer behavior operate similarly across neurocognitive variation. The relevant theoretical issue is therefore not whether influential consumer theories assume a particular cognitive architecture, but whether neurocognitive characteristics constitute meaningful sources of variation in the mechanisms or relationships those theories seek to explain.

A central implication of these developments is the distinction between differences in cognitive performance and differences in cognitive architecture. Here, cognitive architecture refers to the organization and interaction of cognitive processes through which information is attended to, represented, integrated, learned, and translated into behavior. We distinguish this concept from cognitive ability or capacity, which concerns the level or efficiency of performance; cognitive style, which describes relatively consistent preferences in information processing; and cognitive strategy, which refers to the approach adopted to accomplish a particular task. Cognitive architecture therefore concerns the configuration and interaction of underlying processes rather than the quality of an individual’s observed performance on a given task. Traditional psychological models have often interpreted behavioral variation as reflecting differences in ability, motivation, knowledge, or experience operating within a common cognitive system. Recent neuroscientific research, however, increasingly documents individual variation in cognitive pathways, processing strategies, and neural mechanisms that may occur even when individuals achieve similar behavioral outcomes. Consequently, similar consumer behaviors may plausibly arise through different cognitive pathways, implying that identical marketplace outcomes do not necessarily reflect identical cognitive mechanisms.

Attention provides one illustration of this broader theoretical shift. Modern cognitive neuroscience conceptualizes attention as a set of interacting systems responsible for alerting, orienting, sustained attention, and executive control rather than as a single cognitive mechanism (Posner & Petersen, 1990). Individual differences in these systems may influence how consumers allocate cognitive resources, filter competing information, resist distraction, and maintain goal-directed behavior. Consequently, variation in these attentional systems may contribute to differences in how consumers attend to identical marketing stimuli, including among consumers with comparable motivations or product knowledge.

Similar developments have emerged in the study of executive functioning. Executive functions encompass cognitive processes responsible for planning, inhibition, working memory, cognitive flexibility, and self-regulation. Although these mechanisms are central to many theories of consumer decision-making, contemporary research demonstrates considerable variation in executive functioning across individuals and throughout the lifespan (Diamond, 2013; Friedman & Miyake, 2017). Variation in executive functioning may therefore shape how consumers evaluate alternatives, regulate impulses, integrate multiple information sources, and adapt decision strategies in dynamic marketplace environments.

Predictive processing offers another perspective on why cognitive architecture matters for consumer behavior. Recent theoretical models increasingly conceptualize the brain as an active prediction system that continuously generates expectations about incoming sensory information and updates those expectations in response to prediction errors (Friston, 2010; Clark, 2016). Importantly, individuals differ in the extent to which prior expectations or incoming sensory information dominate perception and decision making. Such differences may shape how consumers interpret marketplace signals and respond to uncertainty, ambiguity, novelty, and changing consumption contexts (Wang et al., 2024).

Individual variation is equally evident in reinforcement learning and reward processing. Reinforcement learning and reward processing depend on distributed neural systems, including dopaminergic prediction-error mechanisms that support feedback-based updating and adaptive behavior (Schultz, 2016). Individual differences in reward sensitivity and learning parameters may consequently contribute to heterogeneous responses to promotional incentives, loyalty programs, and repeated marketplace experiences. Consequently, identical marketing interventions may activate different learning processes across consumers, despite producing superficially similar behavioral responses.

Collectively, these developments have contributed to the emergence of the neurodiversity paradigm, which conceptualizes neurodevelopmental variation as a natural dimension of human diversity rather than solely as pathological deviation (Singer, 1999; Doyle, 2020; Pellicano & den Houting, 2022). Although initially associated with autism, the neurodiversity perspective has expanded to encompass a broader range of neurodevelopmental profiles, including attention-deficit/hyperactivity disorder, dyslexia, dyspraxia, Tourette syndrome, and other forms of cognitive variation (Doyle, 2020; Mareva et al., 2024). Central to this perspective is the recognition that differences in cognitive functioning may involve variation in patterns of information processing that cannot always be adequately characterized solely in relation to a single normative standard.

Importantly, adopting a neurodiversity perspective does not require abandoning established scientific distinctions between clinical diagnosis and typical functioning. Terminological precision is important here. Neurodiversity refers to the broader paradigm that recognizes neurological variation as part of human diversity, whereas neurodivergence refers to individuals or profiles that diverge from prevailing neurocognitive norms. In contrast, we use neurocognitive diversity as an analytical concept referring to dimensional variation in cognitive processes across the population, irrespective of diagnostic status. Diagnosed neurodevelopmental conditions may therefore represent one manifestation of neurocognitive variation, but diagnostic categories, neurodivergence, and dimensional neurocognitive characteristics should not be treated as interchangeable constructs. Instead, it encourages a shift in theoretical emphasis from categorizing individuals according to diagnostic labels toward understanding variation in underlying cognitive processes. For consumer research, this distinction is particularly important because marketplace behavior is shaped by cognitive mechanisms rather than diagnostic categories. Consumers do not encounter marketing stimuli as diagnostic groups; they engage with marketplace environments through diverse patterns of perception, attention, learning, and decision making that vary continuously across populations. Importantly, some consumer-specific evidence already suggests that neurocognitive characteristics may be associated with systematic variation in marketplace behavior. For example, research examining autism-spectrum characteristics found that higher Autism-Spectrum Quotient scores were associated with lower levels of exploratory consumer behavior across dimensions including innovativeness, risk taking, exploration through shopping, interpersonal communication, and brand switching (Baas & van Raaij, 2010). Although such evidence does not establish that neurocognitive variation constitutes a general boundary condition across consumer theory, it demonstrates that neurocognitive characteristics can be associated with theoretically relevant differences in consumer behavior and therefore provides an empirical basis for examining these relationships more systematically.

Viewed collectively, these developments document neurocognitive variation as an important feature of human cognition and raise a corresponding question for consumer research: whether such variation constitutes a theoretically meaningful source of consumer heterogeneity. If neurocognitive variation systematically modifies how particular consumer-theory mechanisms operate, then motivations, preferences, identities, and contextual influences may not fully account for heterogeneity in the corresponding consumer outcomes. Neurocognitive diversity may therefore provide an additional dimension for explaining consumer behavior in those theoretical contexts where its incremental effects can be specified and empirically demonstrated. The implications of this shift extend beyond the study of neurodivergent consumers. Rather than introducing a new consumer segment, neurocognitive diversity provides an additional perspective for examining the scope and sources of consumer heterogeneity. The following section builds on this argument by examining whether variation in cognitive architecture may expand prevailing conceptualizations of consumer heterogeneity and by identifying the conditions under which neurocognitive variation may qualify specific consumer-theory predictions.

Heterogeneity has long occupied a central position in consumer research. Much of the discipline’s theoretical progress has been driven by explaining why consumers respond differently to identical marketplace stimuli and by identifying the psychological, social, and contextual factors underlying this variation. Consequently, consumer heterogeneity has traditionally been conceptualized through differences in motivations, preferences, values, attitudes, knowledge, emotions, identities, lifestyles, culture, and situational influences. These dimensions have generated rich theoretical insights and remain fundamental for understanding marketplace behavior.

Despite this extensive body of research, the neurocognitive scope of many established explanations of consumer heterogeneity remains comparatively under-specified. Consumers may differ in what they know, value, believe, or desire, and consumer research has developed sophisticated constructs for explaining such variation. Less explicit, however, is whether and when variation in the cognitive processes through which marketplace information is perceived, processed, integrated, and translated into behavior qualifies these established explanations. The relevant theoretical issue is therefore not that existing approaches necessarily assume a common cognitive architecture, but that neurocognitive characteristics have rarely been articulated as potential scope conditions of particular consumer-theory mechanisms and predictions. Recent advances in cognitive neuroscience provide a basis for investigating this under-specified dimension of consumer heterogeneity. Evidence of systematic individual differences in attentional regulation, executive functioning, predictive processing, cognitive flexibility, and reinforcement learning suggests that neurocognitive characteristics may represent an additional source of behavioral variation. In consumer contexts, such variation may become theoretically consequential when it alters the mechanisms through which marketplace information is encoded, interpreted, evaluated, or translated into action. The key empirical question is therefore whether neurocognitive characteristics explain variation in particular consumer outcomes beyond, or in interaction with, established factors such as motivations, knowledge, preferences, and contextual influences. Recognizing neurocognitive diversity therefore extends, rather than replaces, existing conceptualizations of consumer heterogeneity. Traditional dimensions of heterogeneity remain theoretically indispensable because consumers continue to differ in their goals, experiences, identities, preferences, and social contexts. However, these dimensions may not capture the full range of variation relevant to particular consumer-theory mechanisms and outcomes. Neurocognitive diversity introduces an additional level of analysis by recognizing that the cognitive mechanisms generating consumer responses may themselves differ across individuals.

The theoretical implications become apparent when considering consumers who possess comparable product knowledge, similar demographic characteristics, equivalent purchasing power, and similar preferences within a product category. Models that explain heterogeneity primarily through these characteristics would provide limited grounds for predicting differences between such consumers on the basis of those factors alone. Yet if these consumers differ in attentional regulation, predictive processing, or cognitive flexibility, they may attend to different marketplace cues, construct different interpretations, and ultimately reach different behavioral outcomes despite sharing otherwise comparable psychological characteristics.

Importantly, these differences do not imply that one consumer processes information more effectively than another. Rather, they reflect alternative patterns of cognitive organization through which marketplace information is perceived and interpreted. Consumer behavior therefore emerges not only from differences in motivations, preferences, or decision strategies, but also from differences in the neurocognitive systems that support those strategies. Marketplace behavior can therefore be conceptualized as emerging from interactions between psychological characteristics and neurocognitive processes rather than either dimension in isolation.

This perspective also has important implications for theory development. Reconceptualizing heterogeneity in this way directs additional attention toward specifying whether and under what neurocognitive conditions established theoretical mechanisms and predictions vary. This perspective preserves the explanatory role of existing theories while allowing for the possibility that their explanatory strength may vary across different neurocognitive profiles.

This perspective is consistent with the broader evolution of consumer theory. Throughout its development, the discipline has repeatedly expanded its explanatory scope by incorporating dimensions of variation that were previously overlooked. Models initially emphasizing rational information processing were subsequently extended through research on emotions, experiential consumption, consumer expertise, social identity, culture, and contextual influences. Each extension broadened theoretical understanding without replacing existing foundations. The present framework examines whether incorporating neurocognitive diversity may represent a comparable extension by identifying additional conditions under which established consumer-theory mechanisms and predictions vary.

Importantly, this perspective does not redefine neurodivergent consumers as a distinct market segment requiring separate theoretical treatment. Instead, it draws attention to the extent to which the neurocognitive characteristics of the populations from which consumer theories are derived remain unspecified and to whether such variation affects the generalizability of particular theoretical mechanisms and predictions. Neurocognitive diversity should therefore be understood as a continuum of naturally occurring variation that exists across all consumer populations and interacts with established psychological and contextual determinants of marketplace behavior.

Taken together, these developments raise an important question about the neurocognitive scope of consumer theory. If consumers systematically differ in the organization and functioning of cognitive processes through which marketplace information is processed, such variation may qualify theoretical explanations that focus on established psychological and contextual sources of heterogeneity. A central implication of the framework is that neurocognitive variation should not be conceptualized as producing consumer outcomes independently of the marketplace environment. Rather, its theoretical relevance is expected to emerge through person–environment interactions in which particular neurocognitive characteristics become consequential under specific task, stimulus, or contextual conditions. For example, variation in attentional regulation may become more relevant in environments characterized by high stimulus competition, whereas variation in working-memory or executive processes may become more consequential as information complexity or cognitive demands increase. Neurocognitive characteristics can therefore be conceptualized as potential moderators of established relationships between marketplace conditions, cognitive mechanisms, and consumer outcomes rather than as direct and universally applicable predictors of behavior. The theoretical significance of neurocognitive diversity therefore depends on identifying the specific mechanisms and conditions under which it contributes explanatory value beyond, or in interaction with, these established dimensions. The following section examines this possibility across selected domains of consumer research.

## 5. Extending Consumer Theory Through Neurocognitive Diversity

Building on this argument, the following sections examine how neurocognitive variation may qualify established consumer-theory mechanisms through its interaction with marketplace environments. Consumer theory has traditionally explained behavioral variation through differences in motivations, preferences, knowledge, emotions, identities, and social contexts. These explanations remain fundamental to understanding marketplace behavior. However, if consumers differ systematically in the neurocognitive processes through which they perceive, process, learn from, and evaluate marketplace information, examining such variation may help determine whether established theories leave some mechanism-relevant consumer heterogeneity unexplained.

The following sections focus on four domains—consumer attention, information processing, consumer learning, and judgment and decision making—because each represents a well-established area of consumer research in which theoretical explanations depend directly on cognitive processes that may plausibly vary across individuals. The domains are not intended to provide an exhaustive taxonomy of consumer cognition. Rather, they were selected as theoretically informative cases through which to examine whether neurocognitive variation may qualify established consumer-theory mechanisms and predictions. Across these domains, the analysis identifies potential mechanisms through which neurocognitive variation may interact with marketplace environments and thereby contribute to heterogeneity in consumer outcomes. Accordingly, the framework does not posit neurocognitive variation as uniformly consequential across consumer contexts; rather, it prioritizes conditions in which marketplace environments place comparatively high demands on attentional selection, information processing, feedback-based learning, or decision making. Figure 1 summarizes the proposed framework.

Figure 1 summarizes the proposed framework by positioning neurocognitive variation as a potential scope condition whose theoretical relevance emerges through person–environment interactions. Rather than treating neurocognitive characteristics as direct determinants of consumer behavior, the framework proposes that their effects may depend on characteristics of the marketplace environment, including task demands, stimulus characteristics, information complexity, and digital or contextual demands. These interactions may qualify the cognitive mechanisms underlying consumer attention, information processing, learning, and judgment and decision making, thereby contributing to variation in consumer outcomes and specifying conditions under which established consumer-theory predictions may vary. Consumer attention represents the primary gateway through which marketplace stimuli influence consumer behavior. Whether consumers notice a brand, attend to advertising messages, process product information, or respond to marketplace cues depends fundamentally on how attentional resources are allocated. Consequently, theories of selective attention, information processing, and consumer decision-making have consistently emphasized attention as the mechanism through which marketing stimuli shape subsequent cognitive and behavioral responses (Bettman, 1979; Pieters & Wedel, 2004). Although these theories explain attentional differences through goals, motivations, involvement, prior knowledge, and environmental characteristics, the extent to which neurocognitive variation qualifies the mechanisms governing attentional allocation has received comparatively limited explicit theoretical attention.

Evidence from cognitive neuroscience provides a basis for examining attentional regulation as a potential source of neurocognitive variation relevant to consumer attention. Rather than representing a single cognitive process, attention comprises multiple interacting neural systems responsible for alerting, orienting, sustained attention, and executive control (Posner & Petersen, 1990). Research further demonstrates substantial individual variability in the functioning and coordination of these systems, which may influence how consumers detect, prioritize, and maintain attention toward marketplace information (Corbetta & Shulman, 2002; Petersen & Posner, 2012). Such differences may reflect variation in neurocognitive organization beyond differences associated with motivation, involvement, or task engagement. Importantly, preliminary consumer-specific evidence suggests that such neurocognitive variation may also be observable in marketplace-relevant contexts. Research comparing young consumers with Asperger syndrome with neurotypical controls found significant differences in attentional and emotional responses to advertising stimuli, providing direct evidence that neurocognitive characteristics may be associated with heterogeneous responses to marketing communications (Nuñez-Gomez et al., 2020). Although such findings remain specific to the populations and stimuli examined, they illustrate why the neurocognitive scope of established consumer-attention effects warrants more systematic empirical investigation.

For consumer theory, this shifts the interpretation of attentional differences. Attentional processes reflect not only the characteristics of marketing stimuli, but also the neurocognitive processes through which those stimuli are selected and processed. Two consumers exposed to the same advertisement may attend to different message elements, allocate cognitive resources differently, and construct distinct mental representations despite possessing similar motivations, product knowledge, and purchase intentions. Such differences may arise partly because attentional selection depends not only on stimulus characteristics, but also on the neurocognitive processes involved in filtering, prioritizing, and integrating marketplace information.

Recognizing neurocognitive diversity may therefore extend attention theory by identifying neurocognitive variation as a potential additional source of variation in attentional allocation. Established determinants of attention—including salience, personal relevance, involvement, novelty, and cognitive load—remain fundamental to explaining consumer responses. However, their effects may also vary as a function of neurocognitive characteristics involved in attentional regulation. This possibility suggests that theories of consumer attention could examine whether individual differences in the neurocognitive mechanisms through which marketplace stimuli compete for limited attentional resources qualify established predictions about attentional allocation. This perspective has important implications for contemporary consumer research. Consumers increasingly navigate marketplace environments characterized by continuous information overload across digital, physical, and omnichannel settings. Explaining attentional allocation in such environments may therefore benefit from examining neurocognitive variation alongside established motivational, contextual, and stimulus-related determinants of attention. By incorporating neurocognitive variation as a potential additional dimension of consumer heterogeneity, theories of consumer attention can examine more precisely why identical marketing stimuli may generate different patterns of attentional allocation, and ultimately, different marketplace behaviors among consumers with otherwise comparable psychological characteristics. Accordingly, neurocognitive variation in attentional regulation may be particularly consequential under marketplace conditions characterized by high stimulus competition or attentional demands, where it may moderate the relationship between stimulus characteristics and consumers’ attentional allocation.

## 6. Extending Information Processing Theory

Information processing theory has long provided one of the principal frameworks for explaining how consumers acquire, organize, interpret, store, and use marketplace information (Bettman, 1979; Bettman et al., 1998). Within this tradition, consumer decision making is understood as a sequence of cognitive operations through which external information is transformed into judgments, preferences, and behavioral responses. Existing models primarily explain differences in information processing through variation in motivation, involvement, cognitive capacity, prior knowledge, expertise, and task complexity (Alba & Hutchinson, 1987; Payne et al., 1993). However, the extent to which these information-processing mechanisms and their behavioral consequences vary as a function of neurocognitive characteristics has received comparatively limited explicit theoretical attention.

Evidence from cognitive neuroscience provides a basis for examining whether neurocognitive variation may be relevant to the cognitive processes involved in consumer information processing. Research on working memory and executive functioning indicates meaningful individual variation in the cognitive systems involved in encoding, integrating, and interpreting information (Baddeley, 2000; Diamond, 2013). Predictive-processing accounts further suggest that information processing depends on generative models through which incoming information is interpreted in relation to prior expectations (Friston, 2010; Clark, 2016). Consequently, identical marketplace information may be processed through different cognitive pathways, leading consumers to construct distinct representations of the same consumption environment even when they possess comparable knowledge and motivations.

This evidence adds another layer to traditional information-processing accounts. Divergent consumer responses need not arise only from differences in knowledge, expertise, or decision strategies. A neurocognitively informed perspective suggests that variation may also emerge because consumers differ in the neurocognitive processes through which information is organized, prioritized, and integrated before conscious evaluation begins.

Recognizing neurocognitive diversity also broadens the interpretation of bounded rationality within consumer research. Consumers routinely simplify decisions by relying on heuristics, selective processing, and adaptive strategies when confronted with limited cognitive resources (Payne et al., 1993). Neurocognitive diversity suggests that these adaptive strategies may themselves vary according to differences in attentional regulation, working memory and cognitive flexibility. As a result, consumers who appear to employ similar decision strategies may nevertheless differ in the cognitive processes through which they interpret marketplace information.

The implications are particularly relevant in contemporary digital and omnichannel marketplaces, where consumers are continuously exposed to large volumes of dynamic, multimodal, and often competing information. Under these conditions, neurocognitive variation may influence how consumers filter digital content, navigate online environments, interpret visual interfaces, and integrate information across multiple channels. Considering variation in neurocognitive processes alongside established motivational, knowledge-based, and contextual determinants may therefore help specify when and why consumers process comparable marketplace information differently.

Information-processing theory may therefore be extended by examining neurocognitive variation as a potential additional source of variation in how marketplace information is transformed into judgments and decisions. Future theoretical models can therefore examine whether the same marketplace environment generates different cognitive representations as a function of variation in the neurocognitive processes through which information is perceived, organized, and interpreted. This perspective increases the explanatory precision of information-processing theory while preserving its central insight that consumers actively construct meaning from marketplace information. Accordingly, neurocognitive variation may be particularly consequential under conditions of high information complexity or processing demands, where it may moderate the relationship between the amount and structure of marketplace information and the processes through which such information is encoded, integrated, and interpreted.

## 7. Extending Consumer Learning Theory

Consumer learning theory explains how marketplace experiences shape future judgments, preferences, and behaviors through the acquisition, retention, and application of knowledge (Hoch & Deighton, 1989; Alba & Hutchinson, 1987). Across cognitive, behavioral, and experiential perspectives, learning is generally understood as the process through which consumers accumulate information, recognize patterns, and adapt their behavior over time. Existing theories primarily explain differences in learning through variation in prior knowledge, motivation, repetition, reinforcement, expertise, and environmental feedback (Alba & Hutchinson, 1987; Hoch & Deighton, 1989). However, the extent to which these learning processes and their behavioral consequences vary as a function of neurocognitive characteristics has received comparatively limited explicit theoretical attention.

Evidence from cognitive neuroscience provides a basis for examining neurocognitive variation in the processes underlying consumer learning. Learning depends on the interaction of multiple neurocognitive systems involved in attention, memory, reinforcement learning, and cognitive flexibility (Schultz et al., 1997; Baddeley, 2000; Diamond, 2013). Variation in the functioning of these systems may therefore contribute to individual differences in how marketplace experiences are encoded, retained, and used to guide subsequent behavior. Consequently, consumers may differ not only in how much they learn from marketplace experiences, but also in how information is encoded, organized, retained, and applied. Such differences may partly reflect neurocognitive variation beyond differences associated with motivation, experience, or exposure.

From this perspective, consumer learning involves more than the accumulation of marketplace knowledge; it also depends on the neurocognitive systems through which experience is encoded and transformed into meaningful representations. Consumers repeatedly exposed to identical marketing communications or product experiences may therefore develop different beliefs, expectations, and behavioral tendencies despite possessing similar motivations and receiving equivalent information. Such variation may not be fully captured by differences in experience or prior knowledge alone and may also be associated with variation in the neurocognitive processes supporting learning.

Recognizing neurocognitive diversity also broadens existing theories of experiential learning. Consumer experiences are not passively recorded but actively constructed through perceptual, attentional, and predictive processes that determine how marketplace events are encoded into memory (Friston, 2010; Clark, 2016). Consequently, identical consumption experiences may produce different learning outcomes because consumers differ in how they detect environmental cues, recognize patterns, update expectations, and consolidate new information. Learning therefore reflects an interaction between marketplace experiences and the neurocognitive systems through which those experiences are interpreted.

The implications are particularly relevant in contemporary marketplace environments characterized by personalization, recommendation algorithms, adaptive technologies, and continuous consumer feedback. As consumers repeatedly interact with dynamic digital platforms, neurocognitive variation may influence not only what consumers learn, but also how rapidly they recognize patterns, adapt to changing interfaces, and respond to personalized content. Considering neurocognitive diversity alongside established determinants such as motivation, expertise, and environmental feedback may therefore help specify when and why consumers develop different learning trajectories in otherwise comparable marketplace environments.

Recognizing neurocognitive diversity may extend consumer learning theory by identifying neurocognitive variation as a potential additional source of variation in how marketplace experiences are transformed into knowledge, preferences, and future behavior. Future theoretical models can examine whether identical marketplace experiences generate different learning trajectories as a function of variation in the neurocognitive processes through which experiences are encoded, interpreted, and remembered. This perspective may increase the explanatory precision of consumer learning theory while preserving its central insight that marketplace experiences shape subsequent consumer behavior. Accordingly, neurocognitive variation may be particularly consequential in marketplace contexts involving repeated experience or feedback, where it may moderate the relationship between the structure and timing of marketplace feedback and the processes through which consumers learn from and retain marketplace experiences over time.

## 8. Extending Judgment and Decision-Making Theory

Judgment and decision-making theory represents one of the most influential foundations of consumer research. Existing frameworks explain how consumers evaluate alternatives, construct preferences, assess risks, and make marketplace decisions under conditions of uncertainty and limited information (Kahneman & Tversky, 1979; Bettman et al., 1998; Simonson et al., 2001). Behavioral decision research has further demonstrated that consumer judgments are shaped by heuristics, cognitive biases, contextual framing, affective responses, and bounded rationality, providing a rich understanding of why consumers frequently depart from normative models of rational choice (Tversky & Kahneman, 1974; Kahneman & Tversky, 1979). However, the extent to which the mechanisms supporting judgment and decision making vary as a function of neurocognitive characteristics has received comparatively limited explicit theoretical attention.

Evidence from cognitive neuroscience provides a basis for examining neurocognitive variation in the processes underlying consumer judgment and decision making. Judgment and decision making emerge from the dynamic interaction of multiple neurocognitive systems responsible for attention, executive control, memory, valuation, predictive processing, and emotional regulation (Bechara & Damasio, 2005; Rangel et al., 2008; Karmarkar & Plassmann, 2019). These systems exhibit substantial individual variability that may influence how consumers evaluate information, interpret uncertainty, weigh alternatives, and construct preferences. Consequently, differences in consumer judgments may arise not only from contextual influences or motivational factors, but also from systematic variation in the neurocognitive processes supporting decision making.

This perspective qualifies how variation in consumer choice is interpreted. Preferences, goals, emotions, prior knowledge, and situational context remain important, but they may not fully account for differences in how consumers evaluate alternatives and resolve uncertainty. Variation may also emerge because consumers differ in the neurocognitive processes through which alternatives are evaluated and uncertainty is resolved. Two consumers confronted with the same decision environment may therefore construct different evaluations, assign different subjective values, and ultimately reach different choices despite sharing similar motivations, product knowledge, and contextual circumstances.

Recognizing neurocognitive diversity also broadens the interpretation of heuristics and behavioral biases within consumer research. Heuristics have traditionally been understood as adaptive cognitive shortcuts that simplify complex decisions under conditions of limited information and bounded cognitive resources (Tversky & Kahneman, 1974; Kahneman, 2011). Neurocognitive diversity suggests that the selection, application, and effectiveness of these heuristics may themselves vary according to differences in attentional regulation, executive functioning, predictive processing, and cognitive flexibility. Consequently, consumers who appear to rely on similar decision strategies may nevertheless engage fundamentally different cognitive processes when evaluating marketplace alternatives.

The implications are particularly important in contemporary marketplace environments characterized by algorithmic recommendations, personalized digital interfaces, dynamic pricing, subscription models, and continuously evolving information. Under these conditions, neurocognitive variation may influence not only the outcomes of consumer decisions, but also the processes through which preferences are formed, updated, and maintained over time. Considering neurocognitive diversity alongside established determinants such as framing effects, reference points, involvement, prior knowledge, and affective influences may therefore help specify when and why consumers respond differently to otherwise comparable decision environments.

Recognizing neurocognitive diversity may therefore extend judgment and decision-making theory by identifying neurocognitive variation as a potential additional source of variation in how consumers evaluate information, construct preferences, and make marketplace choices. Future theoretical models can examine whether identical decision environments generate different cognitive processes as a function of variation in the neurocognitive systems through which alternatives are evaluated and uncertainty is interpreted. Such an approach may increase the explanatory precision of judgment and decision-making theory while preserving its central insight that consumer decisions emerge through systematic cognitive processes shaped by marketplace conditions. Accordingly, neurocognitive variation may be particularly consequential under conditions of high choice complexity or uncertainty, where it may moderate the relationship between characteristics of the choice environment and the processes through which consumers evaluate alternatives, construct preferences, and make decisions.

## 9. Toward a Broader Conceptualization of Consumer Heterogeneity

Consumer heterogeneity has long occupied a central position in consumer research. Existing theories recognize that consumers differ in their motivations, goals, preferences, identities, values, knowledge, expertise, emotions, and sociocultural backgrounds, and these differences have provided the foundation for segmentation, targeting, personalization, and theory development across marketing (Wedel & Kamakura, 2000; Wedel & Kannan, 2016). Collectively, this body of research has substantially advanced our understanding of why consumers respond differently to products, brands, marketing communications, and marketplace environments.

Despite these advances, the neurocognitive dimension of consumer heterogeneity remains comparatively under-specified. Existing theories explain variation through differences in what consumers know, value, prefer, or experience, while neurocognitive characteristics are less frequently articulated as potential sources of variation in the processes through which marketplace information is perceived, interpreted, and evaluated. Consequently, the extent to which neurocognitive variation contributes to consumer heterogeneity beyond, or in interaction with, established psychological and contextual determinants remains an open theoretical and empirical question. The preceding sections provide a basis for examining whether neurocognitive variation constitutes a theoretically meaningful additional source of consumer heterogeneity. Evidence from cognitive neuroscience indicates meaningful individual variation in executive functioning and cognitive flexibility (Diamond, 2013; Friedman & Miyake, 2017), the organization of large-scale functional brain networks (Dworetsky et al., 2024), and internal predictive models (Wang et al., 2024). Large-scale neurocognitive systems also support the global availability and flexible routing of consciously processed information (Mashour et al., 2020). These findings document neurocognitive variation at the level of cognitive functioning and organization, while leaving open the consumer-specific question of whether and when such variation explains marketplace outcomes beyond established psychological and contextual sources of heterogeneity.

These directions can be articulated through four research questions corresponding to the core domains of the framework:

RQ1: Under what conditions, particularly those involving high stimulus competition or attentional demands, does neurocognitive variation qualify the effects of established stimulus-, motivation-, and context-related determinants on consumer attentional allocation?

RQ2: Under what conditions, particularly those involving high information complexity or processing demands, does neurocognitive variation qualify how consumers encode, integrate, and interpret comparable marketplace information?

RQ3: Under what conditions, particularly those involving repeated marketplace experiences and differences in the structure or timing of feedback, does neurocognitive variation qualify the relationship between comparable marketplace experiences and subsequent consumer learning and behavior?

RQ4: Under what conditions, particularly those involving high choice complexity or uncertainty, does neurocognitive variation qualify established effects of decision environments on consumer judgment, preference construction, and choice?

Together, these questions translate the proposed framework into empirically testable research directions while leaving the direction and magnitude of neurocognitive effects to empirical investigation. Recognizing neurocognitive diversity may therefore broaden the conceptualization of consumer heterogeneity by identifying neurocognitive characteristics as a potential additional source of variation in marketplace behavior. Traditional sources of variation—including culture, personality, expertise, involvement, motivation, and contextual influences—remain indispensable for explaining marketplace behavior. However, their effects may also depend on the neurocognitive processes through which consumers allocate attention, process information, construct meaning, learn from experience, and make marketplace decisions. Consumer behavior can therefore be conceptualized as emerging from interactions between psychological, social, contextual, and neurocognitive sources of variation rather than any single dimension in isolation.

This broader conceptualization has important implications for theory development. Explicitly recognizing neurocognitive diversity raises the possibility that the scope of particular consumer-theory mechanisms and predictions may vary across neurocognitive characteristics. From this perspective, the neurotypical assumption serves as a heuristic for identifying cases in which neurocognitive scope conditions remain unspecified rather than as an established boundary condition of consumer theory. Making these potential scope conditions explicit enables researchers to examine empirically when existing theoretical predictions generalize across neurocognitive variation and when such variation meaningfully qualifies them.

More broadly, incorporating neurocognitive diversity may extend the explanatory scope of consumer research by providing an additional framework for examining variation in marketplace behavior.

Consumer theory can therefore examine neurocognitive diversity as a potential additional dimension of consumer heterogeneity, whose explanatory relevance depends on whether neurocognitive variation contributes beyond, or interacts with, established psychological, social, demographic, and contextual determinants of consumer behavior. This perspective preserves the explanatory strengths of existing theories while providing a basis for testing whether specifying neurocognitive variation further clarifies their theoretical scope. Table 1 summarizes the principal theoretical extensions proposed in this article and highlights their implications for future consumer research.

## 10. Theoretical and Research Implications

The first theoretical contribution concerns the interpretation and specification of the scope of existing consumer theories. Models of attention, information processing, consumer learning, and judgment and decision making have traditionally explained behavioral variation through differences in motivation, knowledge, preferences, emotions, and contextual influences. The present framework suggests that these explanations may be complemented by examining neurocognitive variation as a potential source of mechanism-relevant heterogeneity. This perspective is consistent with broader developments in management and marketing scholarship emphasizing the importance of explicitly defining the conditions under which theoretical mechanisms are expected to operate (Busse et al., 2017).

The second contribution concerns the conceptualization of consumer heterogeneity itself. Consumer research has traditionally characterized heterogeneity through demographic, psychological, social, and contextual differences (Wedel & Kamakura, 2000). The proposed framework extends this perspective by considering whether neurocognitive diversity may constitute an additional dimension of consumer heterogeneity when neurocognitive variation contributes explanatory value beyond, or in interaction with, established sources of heterogeneity. Consumer behavior may therefore reflect not only differences in what consumers know, value, prefer, or experience, but also variation in the neurocognitive processes through which marketplace information is perceived, interpreted, learned, and translated into behavior. A third contribution is the framework’s potential to strengthen interdisciplinary integration within consumer research. Cognitive neuroscience, developmental psychology, computational cognitive science, and neuropsychology have generated extensive evidence demonstrating systematic variation in attention, executive functioning, predictive processing, reinforcement learning, and cognitive flexibility. This perspective also complements the continuing development of consumer neuroscience by emphasizing theoretical integration rather than methodological expansion alone (Smidts et al., 2014; Karmarkar & Plassmann, 2019).

The proposed framework also identifies several promising directions for future research. Conceptually, future studies should examine whether established consumer phenomena—including framing effects, default effects, choice overload, personalization, advertising effectiveness, and algorithmic recommendations—vary systematically across different patterns of neurocognitive functioning. Such research would extend existing theories by examining the neurocognitive conditions under which established behavioral effects generalize across diverse consumer populations. More broadly, future research can examine whether incorporating neurocognitive variation improves the explanatory precision of models of market segmentation, consumer adaptation, and technology-mediated consumption beyond, or in interaction with, established psychological and contextual sources of heterogeneity.

Beyond its theoretical implications, explicitly recognizing neurocognitive diversity also has important implications for the future development of consumer research and marketing theory. Contemporary marketing increasingly emphasizes market segmentation, personalization, customer experience management, omnichannel design, and inclusive marketing (Lemon & Verhoef, 2016; Wedel & Kannan, 2016; Kannan & Li, 2017; Mende et al., 2024), all of which depend on understanding how consumers perceive, process, and respond to marketplace stimuli. Recent consumer research on disability accessibility further demonstrates that marketplace inclusion is shaped not only by consumer characteristics, but also by how accessibility is incorporated into products, services, and market environments (Grewal & van der Sluis, 2024). This reinforces the importance of examining consumer heterogeneity in relation to the environments with which consumers interact rather than locating difference exclusively within the individual. Rather than treating neurocognitive diversity as relevant only to specific diagnostic groups, the proposed framework highlights neurocognitive variation as a potential source of consumer heterogeneity that may influence how consumers engage with products, services, marketing communications, and digital environments. Methodologically, the framework encourages greater attention to the measurement and operationalization of neurocognitive variability within consumer research.

Future investigations need not rely exclusively on neuroscientific methods. Operationalization should reflect the theoretical level at which neurocognitive variation is expected to matter. Depending on the research question, studies may distinguish between diagnostic-group comparisons, dimensional measures of specific neurocognitive characteristics, and process-based or computational parameters capturing variation in particular cognitive mechanisms. These approaches should not be treated as interchangeable: diagnostic categories may encompass substantial within-group heterogeneity, whereas dimensional measures may provide greater precision when the theoretical prediction concerns a specific cognitive process rather than diagnostic status. Empirical designs should also distinguish neurocognitive variation from correlated characteristics that may provide alternative explanations for observed effects. Relevant considerations include co-occurring conditions, demographic and socioeconomic characteristics, medication use, prior experience, and contextual factors that may influence both cognitive performance and consumer behavior.

Measurement validity is particularly important when constructs developed for clinical or cognitive assessment are transferred to consumer contexts, and researchers should establish that selected measures capture the theoretically relevant neurocognitive process rather than general task performance or adjacent psychological constructs. Where diagnostic groups are examined, analyses should additionally account for substantial heterogeneity within diagnostic categories rather than treating group membership as a proxy for a uniform cognitive architecture. Research on neurocognitive diversity in consumer contexts also requires careful ethical consideration. Identifying neurocognitive characteristics may create risks related to consumer profiling, privacy, discrimination, and the use of cognitive differences to increase persuasive or manipulative targeting. The purpose of incorporating neurocognitive diversity into consumer research should therefore be to improve the theoretical precision and understanding of person–environment interactions rather than to classify consumers into rigid or deficit-based categories. This distinction is consistent with established consumer-vulnerability research, which conceptualizes vulnerability as multidimensional and context dependent and emphasizes that consumer experiences emerge through the interplay of individual characteristics, individual states, and external conditions (Baker et al., 2005). Where research concerns neurodivergent consumers or specific neurodevelopmental conditions, participatory approaches that incorporate lived experience can further help ensure that research questions, measures, and interpretations reflect the perspectives of the populations being studied. Such an approach is particularly important for distinguishing cognitive difference from consumer vulnerability and for avoiding the assumption that variation from prevailing neurocognitive norms necessarily represents impairment. Validated psychological measures, cognitive assessments, behavioral experiments, process-tracing techniques, eye-tracking, pupillometry, computational modeling, and longitudinal research designs all provide promising approaches for testing whether and how variation in neurocognitive functioning is associated with differences in marketplace behavior. Several limitations of the proposed framework should be acknowledged. First, the literature synthesis is purposive and theory-driven rather than systematic or exhaustive; consequently, the present article cannot establish the prevalence of neurocognitive under-specification across consumer research as a whole. Second, much of the evidence reviewed establishes neurocognitive variation in cognitive and neuroscientific contexts rather than directly demonstrating corresponding differences in consumer phenomena. The proposed consumer-theory implications should therefore be understood as theoretically grounded predictions requiring direct empirical validation. Third, the four domains examined—attention, information processing, learning, and judgment and decision making—are illustrative rather than exhaustive, and their incremental explanatory value must be demonstrated relative to established individual-difference, psychological, and contextual constructs.

Moreover, evidence of variation in cognitive performance, capacity, or strategy should not be interpreted as evidence of differences in cognitive architecture unless the organization or interaction of underlying cognitive processes is directly examined.

## 11. Conclusions

Consumer research has long sought to explain why consumers think, feel, and behave differently in marketplace environments. Existing theories have generated profound insights into the roles of motivation, knowledge, culture, identity, emotion, and context in shaping consumer behavior.

Yet the extent to which neurocognitive variation meaningfully qualifies established theoretical mechanisms and predictions concerning how consumers perceive, process, learn from, and evaluate marketplace information remains comparatively under-specified. Advances in cognitive neuroscience increasingly document systematic variation in human cognitive functioning and organization beyond differences in motivation, knowledge, or experience. Viewed from this perspective, neurocognitive variation may constitute an additional dimension of consumer heterogeneity alongside established psychological, demographic, social, and contextual sources of variation. Empirical examination of such neurocognitive scope conditions may therefore help clarify the conditions under which particular consumer-theory mechanisms and predictions generalize. The principal contribution of this article is a conceptual framework for examining whether and under what conditions neurocognitive variation provides explanatory value beyond, or in interaction with, established sources of consumer heterogeneity. As consumer research continues to embrace interdisciplinary perspectives, explicitly recognizing neurocognitive diversity offers new opportunities to refine theory, strengthen empirical inquiry, and develop more comprehensive explanations of consumer behavior.

## Figures and Tables

**Figure 1:**
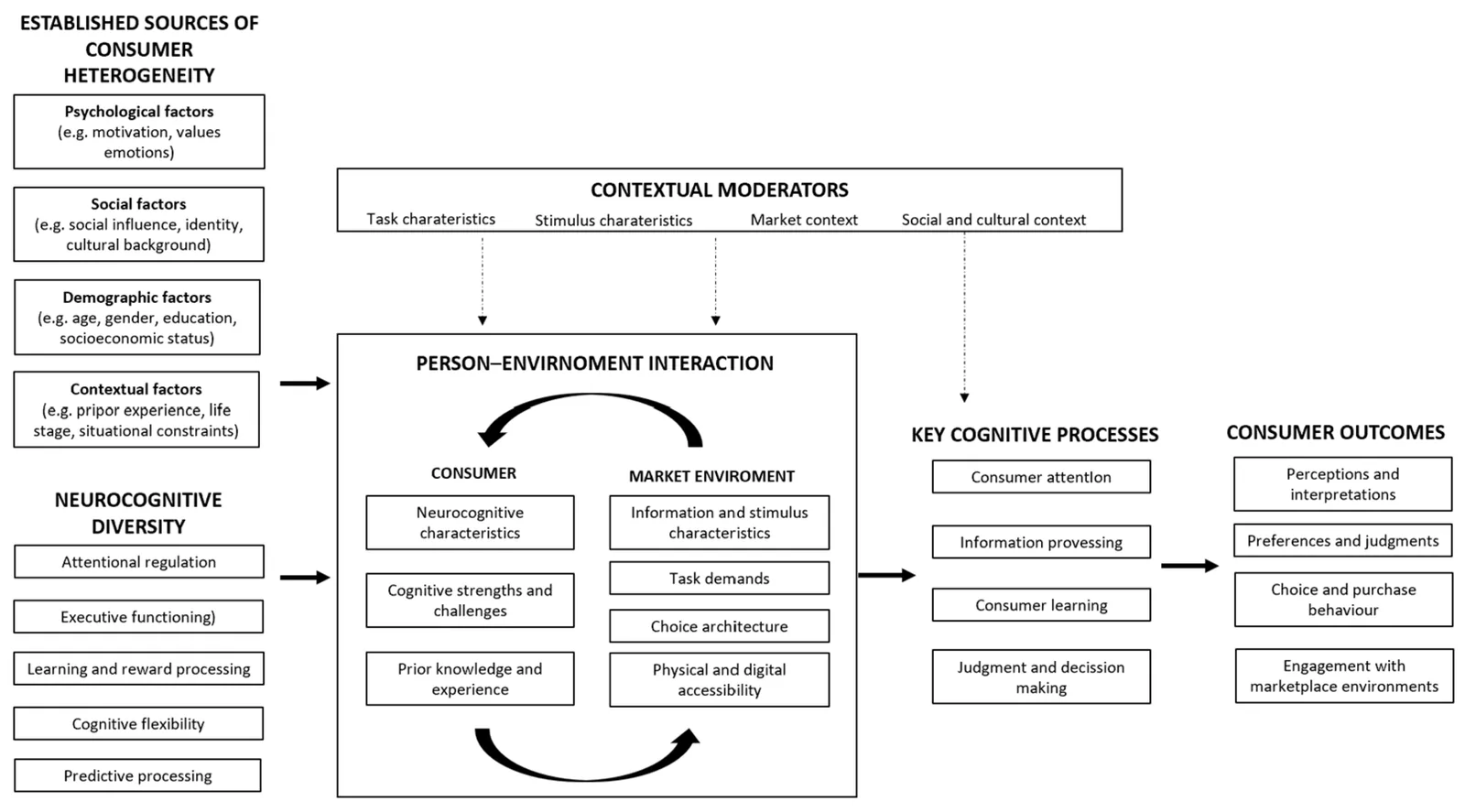
Neurocognitive variation as a potential scope condition in consumer theory. The framework positions neurocognitive diversity alongside established psychological, social, demographic, and contextual sources of consumer heterogeneity. Its theoretical relevance is proposed to emerge through person–environment interactions, whereby neurocognitive characteristics may qualify cognitive mechanisms underlying attention, information processing, learning, and judgment and decision making under particular marketplace conditions, thereby contributing to variation in consumer outcomes.

**Table 1:** Extending consumer theory through neurocognitive diversity.

Traditional Theoretical Domain	Under-Specified Neurocognitive Scope Condition	Theoretical Extension	Implications for Consumer Research
**Consumer attention**	The extent to which neurocognitive variation qualifies attentional mechanisms is rarely specified.	Neurocognitive variation in attentional regulation may qualify patterns of attentional allocation.	Provides testable directions for examining heterogeneity in advertising attention, visual search, and information selection.
**Information processing**	The extent to which neurocognitive variation qualifies information-processing mechanisms is rarely specified.	Neurocognitive variation may qualify the processes through which marketplace information is encoded, integrated, and interpreted.	Provides testable directions for examining variation in digital behavior, interface navigation, and responses to information overload.
**Consumer learning**	The extent to which neurocognitive variation qualifies consumer learning processes is rarely specified.	Neurocognitive variation may qualify the processes through which marketplace experiences are encoded, learned from, and retained over time.	Provides testable directions for examining heterogeneous responses to personalization, feedback, loyalty programs, and repeated marketplace experiences.
**Judgment and decision making**	The extent to which neurocognitive variation qualifies judgment and decision-making mechanisms is rarely specified.	Neurocognitive variation may qualify the processes through which consumers evaluate alternatives, construct preferences, and respond to uncertainty.	Provides testable directions for examining variation in heuristics, framing effects, risk perception, and consumer choice.
**Consumer heterogeneity**	The extent to which neurocognitive variation contributes to consumer heterogeneity beyond established psychological, social, and contextual differences is rarely specified.	Neurocognitive diversity may constitute an additional dimension of consumer heterogeneity when neurocognitive variation contributes explanatory value beyond, or in interaction with, established psychological, social, demographic, and contextual sources of variation.	Provides a basis for testing whether neurocognitive characteristics constitute meaningful boundary conditions for specific consumer-theory predictions.

## Data Availability

No new data were created or analyzed in this study. Data sharing is not applicable to this article.
